# Supplementary material for: Wild vs. Cultivated Zingiber striolatum Diels: Nutritional and Biological Activity Differences
Source: Plants (Basel). 2023 May 31;12(11):2180. doi: 10.3390/plants12112180 (PMC10255660; doi:10.3390/plants12112180)
Supplement: Supplementary file 1 [file plants-12-02180-s001.zip › plants-2411773-supplementary.pdf]

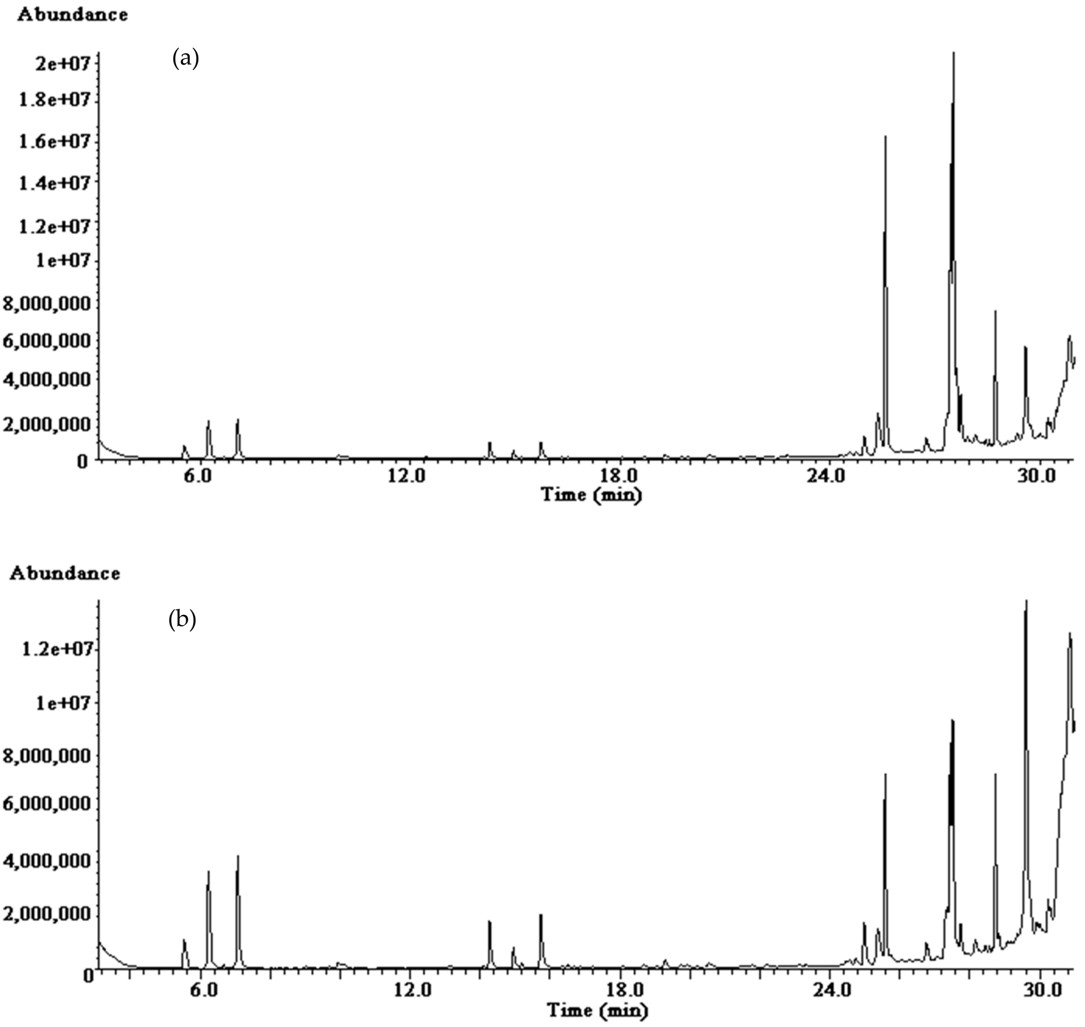

Figure S1. The TICs of wild (a) and cultivated (b) *Zingiber striolatum*.

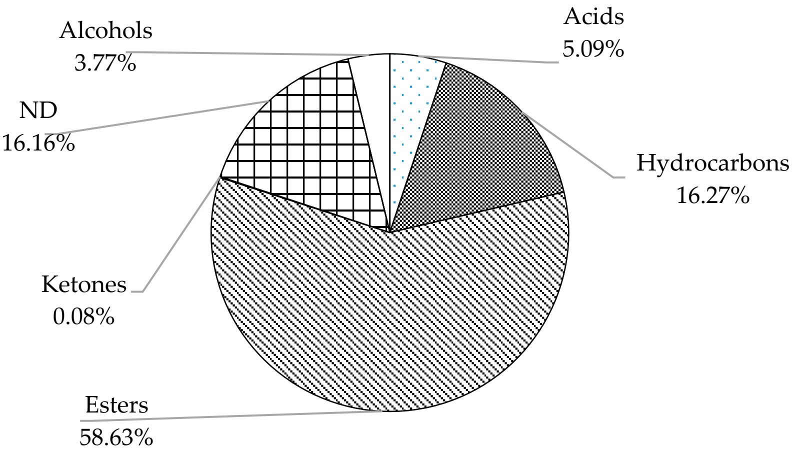

(a)

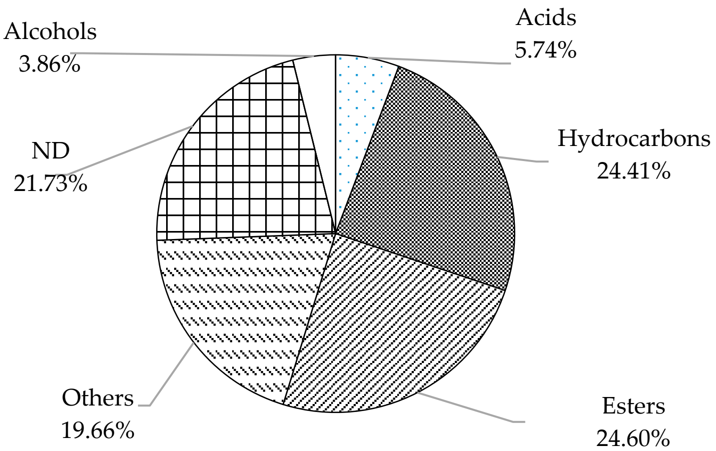

(b)

Figure S2. Charts of the relative content of volatile species in wild (a) and cultivated (b) *Zingiber striolatum.* "ND"means not detected.

Supplementary Materials: The following supporting information can be downloaded at: www.mdpi.com/xxx/s1 , Figure S1: The TICs of wild (a) and cultivated (b) *Zingiber striolatum*. www.mdpi.com/xxx/s12, Figure S2: Charts of the relative content of volatile species in wild (a) and cultivated (b) *Zingiber striolatum.*
